# Cost-effectiveness of Simvastatin plus Ezetimibe for Cardiovascular Prevention in CKD: Results of the Study of Heart and Renal Protection (SHARP)

**DOI:** 10.1053/j.ajkd.2015.09.020

**Published:** 2016-04

**Authors:** Borislava Mihaylova, Iryna Schlackow, William Herrington, Jingky Lozano-Kühne, Seamus Kent, Jonathan Emberson, Christina Reith, Richard Haynes, Alan Cass, Jonathan Craig, Alastair Gray, Rory Collins, Martin J. Landray, Colin Baigent, SHARP Collaborative Group, R. Collins, C. Baigent, M.J. Landray, C. Bray, Y. Chen, A. Baxter, A. Young, M. Hill, C. Knott, A. Cass, B. Feldt-Rasmussen, B. Fellström, D.E. Grobbee, C. Grönhagen-Riska, M. Haas, H. Holdaas, L.S. Hooi, L. Jiang, B. Kasiske, U. Krairittichai, A. Levin, Z.A. Massy, V. Tesar, R. Walker, C. Wanner, D.C. Wheeler, A. Wiecek, T. Dasgupta, W. Herrington, D. Lewis, M. Mafham, W. Majoni, C. Reith, J. Emberson, S. Parish, D. Simpson, J. Strony, T. Musliner, L. Agodoa, J. Armitage, Z. Chen, J. Craig, D. de Zeeuw, J.M. Gaziano, R. Grimm, V. Krane, B. Neal, V. Ophascharoensuk, T. Pedersen, P. Sleight, J. Tobert, C. Tomson

**Affiliations:** 1 Health Economics Research Centre, Nuffield Department of Population Health, University of Oxford, Oxford, United Kingdom; 2 Clinical Trial Service Unit and Epidemiological Studies Unit, Nuffield Department of Population Health, University of Oxford, Oxford, United Kingdom; 3 Menzies School of Health Research, Charles Darwin University, Darwin, Sydney, Australia; 4 School of Public Health, University of Sydney, Sydney, Australia

**Keywords:** Chronic kidney disease (CKD), cardiovascular disease risk, atherosclerotic events, LDL-cholesterol lowering, lipid lowering, cost-effectiveness, statin, ezetimibe, quality-adjusted life-year (QALY), health care costs

## Abstract

**Background:**

Simvastatin, 20 mg, plus ezetimibe, 10 mg, daily (simvastatin plus ezetimibe) reduced major atherosclerotic events in patients with moderate to severe chronic kidney disease (CKD) in the Study of Heart and Renal Protection (SHARP), but its cost-effectiveness is unknown.

**Study Design:**

Cost-effectiveness of simvastatin plus ezetimibe in SHARP, a randomized controlled trial.

**Setting & Population:**

9,270 patients with CKD randomly assigned to simvastatin plus ezetimibe versus placebo; participants in categories by 5-year cardiovascular risk (low, <10%; medium, 10%-<20%; or high, ≥20%) and CKD stage (3, 4, 5 not on dialysis, or on dialysis therapy).

**Model, Perspective, & Timeline:**

Assessment during SHARP follow-up from the UK perspective; long-term projections.

**Intervention:**

Simvastatin plus ezetimibe (2015 UK £1.19 per day) during 4.9 years’ median follow-up in SHARP; scenario analyses with high-intensity statin regimens (2015 UK £0.05-£1.06 per day).

**Outcomes:**

Additional health care costs per major atherosclerotic event avoided and per quality-adjusted life-year (QALY) gained.

**Results:**

In SHARP, the proportional reductions per 1 mmol/L of low-density lipoprotein (LDL) cholesterol reduction with simvastatin plus ezetimibe in all major atherosclerotic events of 20% (95% CI, 6%-32%) and in the costs of vascular hospital episodes of 17% (95% CI, 4%-28%) were similar across participant categories by cardiovascular risk and CKD stage. The 5-year reduction in major atherosclerotic events per 1,000 participants ranged from 10 in low-risk to 58 in high-risk patients and from 28 in CKD stage 3 to 36 in patients on dialysis therapy. The net cost per major atherosclerotic event avoided with simvastatin plus ezetimibe compared to no LDL-lowering regimen ranged from £157,060 in patients at low risk to £15,230 in those at high risk (£30,500-£39,600 per QALY); and from £47,280 in CKD stage 3 to £28,180 in patients on dialysis therapy (£13,000-£43,300 per QALY). In scenario analyses, generic high-intensity statin regimens were estimated to yield similar benefits at substantially lower cost.

**Limitations:**

High-intensity statin-alone regimens were not studied in SHARP.

**Conclusions:**

Simvastatin plus ezetimibe prevented atherosclerotic events in SHARP, but other less costly statin regimens are likely to be more cost-effective for reducing cardiovascular risk in CKD.

SHARP (Study of Heart and Renal Protection) has shown that lowering low-density lipoprotein (LDL) cholesterol levels by 0.85 mmol/L with a combination of simvastatin, 20 mg, plus ezetimibe, 10 mg, daily (simvastatin plus ezetimibe) reduces the risk of major atherosclerotic events in a broad range of patients with moderate to severe chronic kidney disease (CKD), without adverse effects of treatment.^1^ The KDIGO (Kidney Disease: Improving Global Outcomes) clinical practice guideline for lipid management in CKD recommends that treatment with a statin, or a statin plus ezetimibe, be initiated in all non–dialysis-dependent and nontransplantation patients with CKD who are 50 years or older, as well as in younger patients with elevated cardiovascular risk.^2^ In the United Kingdom, the National Institute for Health and Care Excellence recommends atorvastatin, 20 mg, daily (available generically) for all patients with CKD.^3^ Similarly, the 2013 American College of Cardiology and American Heart Association guidelines recommend statin therapy for non–dialysis-dependent patients with CKD who are at increased risk for cardiovascular disease.^4^

Although SHARP provided clear evidence that simvastatin plus ezetimibe is effective for the prevention of major atherosclerotic events in patients with CKD, little information is available about its cost-effectiveness.^5^ We therefore assess the cost-effectiveness of simvastatin, 20 mg, plus ezetimibe, 10 mg, daily during the study scheduled treatment period and the extent to which its cost-effectiveness varies in relation to the cardiovascular risk of such patients, the severity of kidney disease, and the cost of treatment. The relevance of other high-intensity statin regimens is investigated in scenario analyses.

## Methods

### Study Design

Details of the SHARP design and its main results have been reported previously.^1^, ^6^ SHARP was registered at ClinicalTrials.gov (study number: NCT00125593). Patients 40 years or older with CKD but without known coronary heart disease were eligible if they were receiving maintenance dialysis or had serum or plasma creatinine levels of at least 150 μmol/L (1.7 mg/dL) in men or 130 μmol/L (1.5 mg/dL) in women. Participants randomly assigned to simvastatin plus ezetimibe versus placebo (Fig S1, available as online supplementary material) were to be seen in the study clinics at least once every 6 months for at least 4 years. Adherence to study treatment was assessed at each follow-up, and the use of any nontrial lipid-lowering medication was recorded. Central laboratory assays of lipid profiles were conducted in all study participants at randomization and at the 2.5-year visit. At each follow-up visit, information was recorded about any suspected myocardial infarction, stroke, vascular procedure, cancer, hospital admission for other reasons, or other serious adverse event. Further information was sought from the participant, his or her physician, or hospital records and other sources.

Because the cost-effectiveness of treatments that reduce LDL cholesterol levels in other populations is strongly determined by the cardiovascular risk of such populations,^7^ we developed a Cox proportional hazards risk-prediction model^8^ to categorize SHARP patients according to whether their 5-year risk for cardiovascular events (defined as nonfatal myocardial infarction, nonfatal stroke, any revascularization procedure, or vascular death) was low (<10%), medium (10%-<20%), or high (≥20%; Table S1).

### Assessment of Health Outcomes and Health Care Costs

The cost-effectiveness analysis is performed for the period from randomization (to simvastatin plus ezetimibe vs placebo) until the final study visit (ie, 4.9 years’ median follow-up). The prespecified health outcome in the cost-effectiveness analysis was major atherosclerotic event (myocardial infarction or death from coronary heart disease, nonhemorrhagic stroke, or arterial revascularization excluding dialysis access procedures), the key end point in SHARP.^1^, ^9^ The cost-effectiveness analysis was developed from the perspective of the UK National Health Service (NHS), and all calculations are based on applying UK NHS costs to health care resource use recorded in the study. All major atherosclerotic events and other serious adverse events recorded during the study (ie, not just the first event of any particular type) were included. To estimate the cost of hospital care, hospital episodes were mapped onto UK Healthcare Resource Groups or onto hospital specialty for some outpatient episodes, with costs in 2011 UK pounds (£). Hospital episodes were classified into 1 of 4 categories: (1) atherosclerotic (including coronary, cerebrovascular, revascularization, and other vascular atherosclerotic), (2) nonatherosclerotic vascular, (3) renal, or (4) other (nonvascular and nonrenal). When a hospital episode involved serious adverse events in more than 1 category, the episode was classified hierarchically according to the above mentioned order. The cost of routine dialysis was calculated based on dialysis status and modality recorded for participants during the study, using the UK recommended frequency of dialysis sessions^10^ and average cost per session.^11^

Participants allocated to simvastatin plus ezetimibe who were recorded at a scheduled follow-up as having taken ≥10% of their allocated study treatment were attributed a daily simvastatin plus ezetimibe cost of £1.19 (2015 UK price)^12^ over that follow-up period. It was assumed that participants taking <10% of the medication would not obtain a repeat prescription in ordinary clinical practice, so no cost was attributed to such patients while adherence remained at that level. Use of nonstudy medication, by category, was compared between treatment arms at 2.5 years using Pearson χ^2^ test. To improve the precision of cost-effectiveness estimates, only categories of hospital episodes and other health care use for which there were statistically significant effects of allocation to study treatment were included in cost-effectiveness analyses.

### Cost-effectiveness Analyses

Intention-to-treat analyses assessed the effects of allocation to simvastatin plus ezetimibe on major atherosclerotic events, rate of hospital episodes, and costs of hospital episodes for the duration of follow-up among all patients with CKD randomly assigned to simvastatin plus ezetimibe versus placebo in SHARP, as well as in patient categories by 5-year cardiovascular risk and CKD stage (3, 4, 5 not on dialysis, or on dialysis therapy). Quasi-Poisson models with sandwich standard errors^13^ were used to estimate rate ratios (RRs) for different outcomes, and standard χ^2^ tests for heterogeneity or trend were used to examine differences in RRs between different categories of patients. In randomized trials of statin regimens^14^, ^15^ and ezetimibe,^16^ the proportional reductions in major atherosclerotic events achieved by different regimens are proportional to the absolute reduction in LDL cholesterol level achieved. Because the LDL cholesterol level reduction achieved with simvastatin plus ezetimibe in specific patient subgroups in SHARP was influenced by baseline LDL cholesterol level in that subgroup, as well as the degree of adherence to allocated treatment and use of nonstudy statin therapy,^1^ RRs for each outcome were adjusted for such differences and are presented as RRs per 1 mmol/L of LDL cholesterol reduction (as measured at the study midpoint). In the absence of statistically significant heterogeneity of the effects of treatment on major atherosclerotic events per 1-mmol/L reduction in LDL cholesterol level across categories of participants, the absolute reduction in such events in a particular subgroup was derived by applying the overall proportional reduction in this outcome (ie, among all SHARP participants) corresponding to the achieved LDL cholesterol reduction in the respective subgroup to the rate among placebo-allocated patients in that subgroup. Subgroup-specific absolute reductions in vascular hospital episode costs were calculated in an analogous way. The cost of simvastatin plus ezetimibe for a particular subgroup was based on the level of use required to achieve the observed LDL cholesterol level reduction in that subgroup. The net cost of treatment for each subgroup was calculated by subtracting the estimated hospital cost savings from the cost of simvastatin plus ezetimibe. Division of these net costs by the absolute reduction in major atherosclerotic events yielded cost-effectiveness estimates (net costs per major atherosclerotic event avoided).

Cost-effectiveness results are presented for the reductions in LDL cholesterol levels actually observed in SHARP (ie, incorporating actual nonadherence and use of nonstudy statin treatment). Future costs and outcomes were discounted at 3.5% per annum.^17^ A bootstrap procedure was used to evaluate the stochastic uncertainty in cost-effectiveness results,^18^ and the 95% confidence intervals (CIs) for events avoided, additional costs, and cost-effectiveness were evaluated using the percentile method.

### Sensitivity Analyses

Sensitivity analyses assessed the cost-effectiveness of simvastatin plus ezetimibe treatment based on treatment costs ranging from the current UK simvastatin plus ezetimibe price of £1.19 per day to the cost of a UK generic statin regimen that produces a similar proportional reduction in LDL cholesterol level (eg, £0.05-£0.10 per day^12^; Table S2). Cost-effectiveness was also assessed under an assumption of full adherence with treatment. The net costs per major vascular event (major atherosclerotic event, noncoronary cardiac death [eg, nonischemic heart failure, valvular heart disease, and arrhythmic cardiac death], or hemorrhagic stroke) avoided were also calculated. Finally, the cost-effectiveness of simvastatin plus ezetimibe among dialysis patients was also evaluated using the point estimate for such patients in SHARP because meta-analyses suggest that the effects of LDL cholesterol lowering in a dialysis population may be smaller than in other patients with CKD.^19^, ^20^, ^21^

### Long-term Projections

To assess the effects of avoiding major atherosclerotic events during SHARP on quality-adjusted survival and end-stage renal disease (ESRD) costs, separate Gompertz proportional hazards parametric survival models were fitted to participants having and not having major atherosclerotic events during SHARP (matched by their 5-year estimated cardiovascular risk^22^), and median overall survival was then calculated by cardiovascular risk and CKD stage category. The differences between these survival times provided the projected gains in survival due to avoiding a major atherosclerotic event. Quality of life during projected survival was based on stage of CKD,^5^ and ESRD costs were calculated using rates of progression to ESRD by CKD stage estimated in SHARP^23^ and assuming a 2:1 ratio between dialysis and transplantation and annual ESRD costs.^24^

### Other High-Intensity Statin Regimens

Although the effectiveness of regimens other than simvastatin plus ezetimibe was not assessed directly in SHARP, we conducted scenario analyses with other high-intensity statin regimens that can achieve a 40% or higher LDL cholesterol reduction.^3^ Table S2 summarizes the reductions in LDL cholesterol level achieved with such regimens and their UK daily cost. Based on the known linear relationship between mean absolute LDL cholesterol level reduction and risk reduction in a wide range of patients in a meta-analysis of large statin trials^14^ and in a trial of ezetimibe added to a statin regimen,^16^ we calculated the likely reductions in major atherosclerotic events with different regimens by scaling the proportional reduction observed in SHARP by the ratios of proportional reductions in LDL cholesterol level achieved (Table S2).

Further detail on methods is available in Item S1.

## Results

### Effects of Randomized Allocation to Simvastatin plus Ezetimibe in SHARP

**Figure 1:**
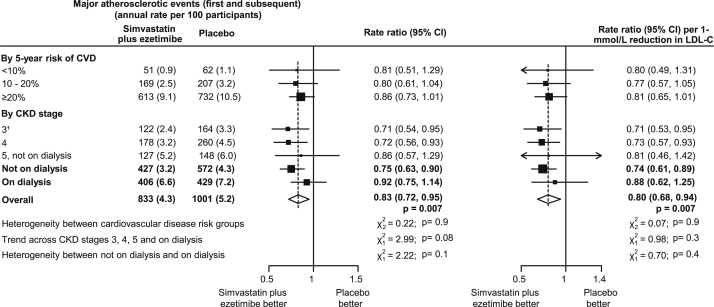
Effect of allocation to simvastatin plus ezetimibe treatment on all major atherosclerotic events in SHARP (Study of Heart and Renal Protection). ^1^83% of participants in this category with chronic kidney disease (CKD) stage 3b (estimated glomerular filtration rate of 30-<45 mL/min/1.73 m^2^). Abbreviations: CI, confidence interval; CVD, cardiovascular disease; LDL-C, low-density lipoprotein cholesterol.

Overall, 9,270 patients from 18 countries were randomly assigned to simvastatin plus ezetimibe (4,650 patients) versus placebo (4,620 patients) in SHARP. Baseline characteristics of participants, subdivided by estimated cardiovascular risk and CKD stage, are shown in Table 1. Allocation to simvastatin plus ezetimibe produced a mean reduction of 0.85 mmol/L in LDL cholesterol level, which yielded a 17% proportional reduction (rate ratio [RR], 0.83; 95% CI, 0.72-0.95; *P* = 0.007) in all (first and subsequent) major atherosclerotic events, corresponding to a 20% proportional reduction (RR, 0.80; 95% CI, 0.68-0.94) per 1-mmol/L LDL cholesterol level reduction (Fig 1). After allowing for differences in LDL cholesterol levels achieved (Table S3), there was no significant heterogeneity of this reduction across categories by cardiovascular risk (*P* for heterogeneity = 0.9) and no significant trend toward smaller proportional reductions with more severe CKD stage (*P* for trend = 0.3; Fig 1).

**Figure 2:**
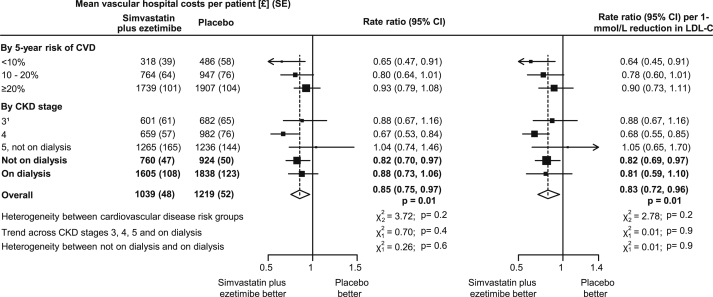
Effect of allocation to simvastatin plus ezetimibe treatment on costs of vascular hospital episodes in SHARP (Study of Heart and Renal Protection). ^1^83% of participants in this category with chronic kidney disease (CKD) stage 3b (estimated glomerular filtration rate of 30-<45 mL/min/1.73 m^2^). Abbreviations: CI, confidence interval; CVD, cardiovascular disease; LDL-C, low-density lipoprotein cholesterol; SE, standard error.

Overall, allocation to simvastatin plus ezetimibe yielded a 16% proportional reduction (RR, 0.84; 95% CI, 0.74-0.96; *P* = 0.01) in atherosclerotic hospital episodes and an 11% reduction (RR, 0.89; 95% CI, 0.79-0.99; *P* = 0.04) in nonatherosclerotic vascular episodes (Table S4). These effects resulted in a 15% proportional reduction (RR, 0.85; 95% CI, 0.75-0.97; *P* = 0.01) in mean costs of all vascular hospital episodes (corresponding to 17% proportional reduction [RR, 0.83; 95% CI, 0.72-0.96] per 1-mmol/L LDL cholesterol reduction), with no significant heterogeneity of the proportional reduction in costs among subgroups defined by 5-year risk of cardiovascular disease (*P* for heterogeneity = 0.2) or by CKD stage (*P* for trend = 0.9; Fig 2). The number of vascular hospital episodes (ie, episodes that included any vascular event) by case-mix group or specialty and the respective unit costs are presented in Table S5.

Allocation to simvastatin plus ezetimibe had no significant effect on renal hospital episodes (RR, 0.97; 95% CI, 0.90-1.03; *P* = 0.3) or other nonvascular and nonrenal hospital episodes (RR, 1.03; 95% CI, 0.97-1.09; *P* = 0.4; Table S4), so the costs of both were excluded from subsequent calculations of cost-effectiveness. Similarly, there were no significant differences in the costs of routine dialysis or in the use of medications other than nonstudy lipid-lowering medications, so these costs were also excluded.

The mean cost per patient of simvastatin plus ezetimibe required to achieve the LDL cholesterol level reduction actually observed in SHARP during the study follow-up was £1,319 ± £11 (standard error; Table 2). There was a trend toward lower costs of treatment among those at higher 5-year risk for cardiovascular disease. This was attributable to shorter duration of treatment among those at higher risk for death; for example, in the highest and lowest risk groups, the costs of study simvastatin plus ezetimibe were £1,137 ± £16 and £1,570 ± £20, respectively, reflecting mean treatment durations of 1,038 ± 15 and 1,400 ± 18 days, respectively.

Because CKD stage and estimated cardiovascular risk were correlated (Table 1), the cost per patient was lowest among those with more severe CKD (£1,182 ± £29 among those at stage 5 not on dialysis therapy and £1,214 ± £20 among those on dialysis therapy) and highest among those with CKD stage 3 (£1,454 ± £20).

### Base-Case Cost-effectiveness Results

Among all randomly assigned patients, 37 major atherosclerotic events were avoided per 1,000 participants allocated to simvastatin plus ezetimibe over about 5 years, and the net cost of treatment to achieve the reduction in LDL cholesterol levels observed in SHARP (ie, simvastatin plus ezetimibe cost minus vascular hospital episode cost saving) was £1,142 (95% CI, £1,004-£1,282); the overall cost-effectiveness of simvastatin plus ezetimibe was £30,390 per major atherosclerotic event avoided (Table 3). Taking into account the absolute rates of major atherosclerotic events and costs of vascular hospital episodes among placebo-allocated participants across the participant subgroups, the reduction in major atherosclerotic events per 1,000 participants ranged from 10 in low-risk to 58 in high-risk patients and from 28 in patients with CKD stage 3 to 36 in patients on dialysis therapy at randomization. The net cost of avoiding a major atherosclerotic event ranged from £157,060 (95% CI, £84,090-£597,940) among patients at low cardiovascular risk to £15,230 (95% CI, £7,220-£64,410) among patients at high risk (Table 4) and from £47,280 (95% CI, £26,370-£173,760) among patients in CKD stage 3 to £28,180 (95% CI, £13,820-£115,380) among patients on dialysis therapy. Similar results were obtained in analyses of major vascular events (Fig S2; Table S6).

### Sensitivity Analyses

**Figure 3:**
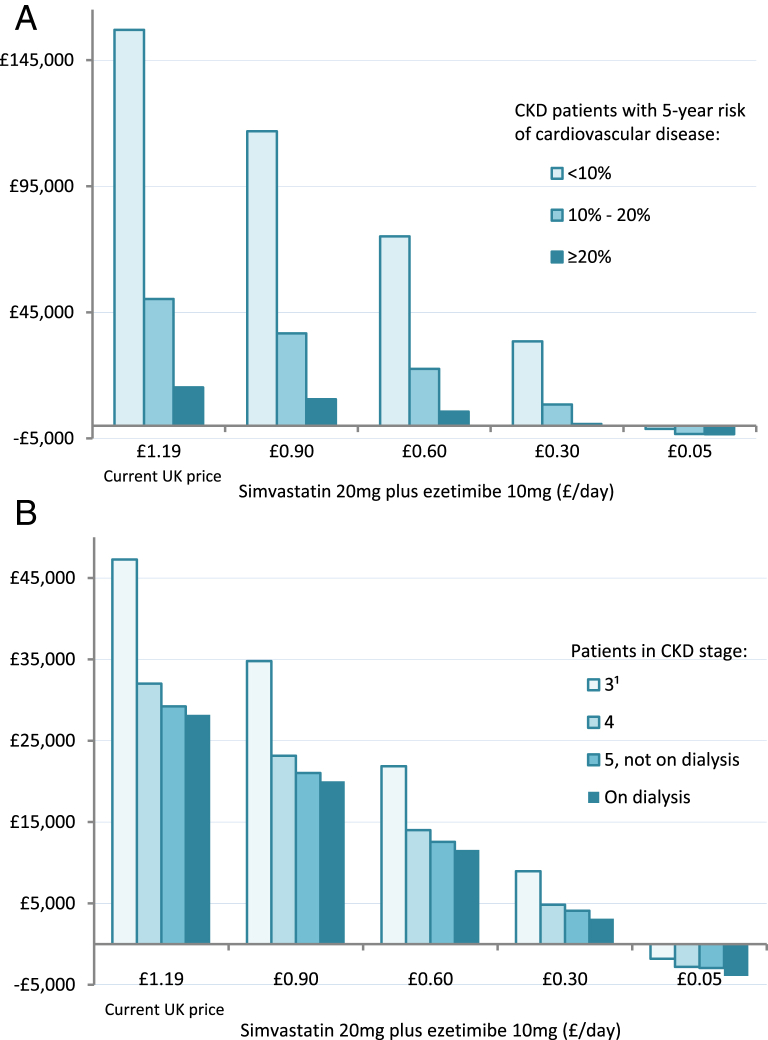
Net cost per major atherosclerotic event avoided in SHARP (Study of Heart and Renal Protection) with simvastatin, 20 mg, plus ezetimibe, 10 mg, daily at different prices in patients (A) at different cardiovascular disease risk and (B) at different stages of chronic kidney disease (CKD). Negative figures indicate cost savings. ^1^83% of participants in this category with CKD stage 3b (estimated glomerular filtration rate of 30-<45 mL/min/1.73 m^2^).

The cost-effectiveness of simvastatin plus ezetimibe was very sensitive to drug price (Fig 3), with net cost per major atherosclerotic event decreasing nearly linearly with lower price. Full adherence with simvastatin plus ezetimibe would achieve larger treatment benefits but would also incur additional treatment costs, so full adherence would result in only a slight reduction in incremental cost-effectiveness ratios (Tables S6 and S7).

Calculation of the cost-effectiveness of simvastatin plus ezetimibe among dialysis patients, based on the point estimate for the treatment effect among dialysis patients only (ie, 8% [95% CI, −14% to 25%] reduction in major atherosclerotic events and 12% [95% CI, −6% to 27%] reduction in costs of vascular hospital episodes; Figs 1 and 2) produced a nonsignificant 22 (95% CI, −38 to 80) major atherosclerotic events avoided per 1,000 at a net cost per event avoided of £45,880, with the 95% CI spreading both across areas of net health benefit and harm.

### Net Cost per Quality-Adjusted Life-Year

Estimated differences in median survival due to avoiding a major atherosclerotic event ranged from 12 years (8.9 quality-adjusted life-years [QALYs]) among those at low cardiovascular risk to 1.9 years (1.4 QALYs) among those at high risk and from 7.1 years (6.2 QALYs) among those in CKD stage 3 to 3.4 years (2.4 QALYs) among those in receipt of dialysis therapy at randomization. During this added survival time, additional ESRD costs in the range of £23,500 to £154,300 across vascular risk groups and £20,800 to £54,100 across CKD stages were projected. In the base-case cost-effectiveness analysis, the use of simvastatin plus ezetimibe for about 5 years in SHARP is projected to result in net costs of £30,500 to £39,600 per QALY across risk groups and £13,000 to £43,300 per QALY across CKD stages (Tables 4 and S8).

### Scenario Analyses of Relevance of Other High-Intensity Statin Regimens

For scenario analyses, we assumed that high-intensity statin regimens other than simvastatin plus ezetimibe would achieve effects on major atherosclerotic events and hospital costs that were in proportion to their relative potency in reducing LDL cholesterol levels compared to simvastatin plus ezetimibe (see Methods and Table S2), and that they would not result in additional adverse effects. The net cost per major atherosclerotic event avoided with such a regimen would be similar to simvastatin plus ezetimibe if it were available at identical cost (Fig 3). Table 4 summarizes the projected net cost per QALY for high-intensity statins at different costs. For example, with atorvastatin, 20 mg, daily (£0.05 per day), the net cost per QALY would be £12,700 to £17,300 across the vascular risk categories and £3,100 to £20,100 across CKD stages (Table 4).

## Discussion

Compared to no LDL cholesterol–lowering therapy, the net cost per major atherosclerotic event avoided among SHARP patients with about 5 years of treatment with simvastatin plus ezetimibe at a UK 2015 cost of £1.19 per day ranged from £15,230 in those at high risk for cardiovascular disease to £157,060 in those at low risk (about £30,500-£39,600 per QALY gained) and from £47,280 in those with CKD stage 3 to £28,180 in those on dialysis therapy (£13,000-£43,300 per QALY). The Improved Reduction of Outcomes: Vytorin Efficacy International Trial (IMPROVE-IT) trial of simvastatin plus ezetimibe versus simvastatin alone among patients with acute coronary syndromes^16^ has shown that the benefit of ezetimibe on major atherosclerotic events is equivalent to that which can be achieved by the same LDL cholesterol level reduction with a statin.^14^ Therefore, the benefits observed in SHARP are likely to be possible using a statin regimen that produces a comparable LDL cholesterol level reduction (as long as such a regimen does not yield additional adverse effects). Our scenario analyses using SHARP data and modeling use of alternative high-intensity generic statin regimens at a UK 2015 cost of £0.05 to £0.10 per day suggested that they would be the most cost-effective option for patients with CKD. Although the costs of generic statin regimens might be greater in other countries (eg, generic atorvastatin, 20 mg, available at $0.15 per day in the United States^25^), it is still likely at present that such regimens, when available, will be a more cost-effective means than simvastatin plus ezetimibe ($6.92 per day in the United States^25^) for reducing cardiovascular risk in patients with CKD.

One would expect the observed decrease in vascular events to also affect primary care and outpatient resource use, but these data were not collected systematically in SHARP. Nevertheless, the expense of those services would probably be small in comparison to the hospital costs and unlikely to materially affect the estimates presented here.^26^ In SHARP, simvastatin plus ezetimibe did not have statistically significant effects on costs related to nonvascular events, to non–lipid lowering concomitant medications, or to routine maintenance dialysis during the study, so these costs were excluded in order to improve the precision of cost-effectiveness estimates. Likewise, the costs of laboratory monitoring (and staff time reviewing laboratory findings) were also not included in these cost-effectiveness analyses because routine monitoring of lipid levels and safety (eg, for creatine kinase or hepatic enzyme elevations) is not required for statin-based treatments.^2^, ^4^ Moreover, because patients with moderate to severe CKD are seen regularly in the clinic, any services related to the prescription of lipid-lowering medications are likely to take place in the context of a routine consultation. Estimated professional dispensing fees equate to ∼£0.02 per day,^12^ so dispensing costs would also have had minimal effect on the cost-effectiveness estimates. Hospital resource use may have varied across the 18 countries that participated in SHARP, but because the present analyses use randomized comparisons of hospital episodes and UK Healthcare Resource Groups costs to value them, this is unlikely to have materially affected our results. Finally, meta-analyses of randomized trials conducted in a broad range of patients, including those at high risk for cardiovascular disease, indicate that the absolute excess risks of the known or probable adverse effects of statin therapy (specifically myopathy, new-onset diabetes mellitus, and hemorrhagic stroke) are substantially smaller than the absolute benefits, so the impact of adverse effects on cost-effectiveness is likely to be small.^27^

Because SHARP did not provide definite evidence that the proportional effects of reducing LDL cholesterol levels varied with initial CKD stage, the cost-effectiveness of simvastatin plus ezetimibe among dialysis patients was estimated using the treatment effect estimated among all SHARP patients (ie, a 17% proportional reduction in major atherosclerotic events and 15% reduction in vascular hospital costs). However, several meta-analyses have suggested that the proportional effects of treatment may be smaller among dialysis patients.^19^, ^20^, ^21^ If the true effects of simvastatin plus ezetimibe (or other high-intensity statin regimens) among dialysis patients on such outcomes are similar to the subgroup-specific effect observed in SHARP, somewhat smaller benefits could be expected at higher net cost per event avoided with a much wider CI.

The cost-effectiveness estimates in the present report are for treatment health effects and treatment costs just within a 5-year period, but longer term treatment would yield larger benefits. Quantification of such gains requires allowance for longer term use and cost of interventions and effect over time of all renal and cardiovascular events, as well as for increasing age, because the rapid increase in vascular disease risk with older age is offset by a decrease in life expectancy. Further analyses of SHARP using a simulation model are examining the cost-effectiveness of long-term use of statin-based regimens.

## Figures and Tables

**Table 1:** Baseline Characteristics of SHARP Participants by CVD Risk and CKD Stage at Randomization

	Overall N = 9,270	By 5-year Risk of CVD			By CKD Stage			
<10% (n = 2,489)	10%-<20% (n = 2,997)	≥20% (n = 3,784)	3^a^ (n = 2,323)	4^b^ (n = 2,663)	5^c^ (n = 1,259)	On dialysis (n = 3,025)
Age, y	62 ± 12	52 ± 8	61 ± 11	69 ± 10	63 ± 12	63 ± 12	62 ± 12	60 ± 12
Male sex	5,800 (63)	1,206 (48)	1,915 (64)	2,679 (71)	1,691 (73)	1,562 (59)	629 (50)	1,918 (63)
Current smoker	1,243 (13)	237 (10)	443 (15)	563 (15)	299 (13)	330 (12)	142 (11)	472 (16)
Previous vascular disease	1,393 (15)	44 (2)	173 (6)	1,176 (31)	343 (15)	400 (15)	189 (15)	461 (15)
Diabetes mellitus	2,091 (23)	106 (4)	388 (13)	1,597 (42)	557 (24)	611 (23)	255 (20)	668 (22)
Body mass index, kg/m²	27.1 ± 5.6	26.9 ± 5.5	27.1 ± 5.6	27.2 ± 5.7	27.7 ± 5.1	27.5 ± 5.7	26.5 ± 5.4	26.5 ± 5.9
Cholesterol								
Total, mmol/L	4.89 ± 1.18	5.05 ± 1.08	4.91 ± 1.14	4.76 ± 1.26	5.05 ± 1.13	5.09 ± 1.15	4.79 ± 1.24	4.63 ± 1.17
HDL, mmol/L	1.12 ± 0.34	2.89 ± 0.82	2.78 ± 0.86	2.69 ± 0.91	1.14 ± 0.34	1.14 ± 0.34	1.11 ± 0.33	1.08 ± 0.35
LDL, mmol/L	2.78 ± 0.87	1.13 ± 0.33	1.13 ± 0.35	1.06 ± 0.33	2.91 ± 0.85	2.92 ± 0.85	2.69 ± 0.9	2.58 ± 0.86
Triglycerides, mmol/L	2.32 ± 1.72	2.26 ± 1.47	2.34 ± 1.57	2.35 ± 1.97	2.43 ± 1.95	2.35 ± 1.45	2.09 ± 1.43	2.32 ± 1.85
Diastolic BP, mm Hg	79 ± 13	82 ± 12	80 ± 13	77 ± 13	80 ± 13	80 ± 13	80 ± 12	78 ± 13
Systolic BP, mm Hg	139 ± 22	132 ± 18	138 ± 21	144 ± 23	139 ± 20	139 ± 21	141 ± 21	138 ± 24
5-year risk of CVD								
<10%	2,489 (27)				995 (43)	873 (33)	285 (23)	336 (11)
10%-<20%	2,997 (32)				769 (33)	915 (34)	366 (29)	947 (31)
≥20%	3,784 (41)				559 (24)	875 (33)	608 (48)	1,742 (58)

*Note:* Values for categorical variables are given as number (percentage); for continuous variables, as mean ± standard deviation.

Abbreviations: BP, blood pressure; CKD, chronic kidney disease; CVD, cardiovascular disease; eGFR, estimated glomerular filtration rate; HDL, high-density lipoprotein; LDL, low-density lipoprotein; SHARP, Study of Heart and Renal Protection.

a eGFR ≥ 30 mL/min/1.73 m^2^. 83% of participants in this category with CKD stage 3b (eGFR 30-45 mL/min/1.73 m^2^).

b eGFR of 15 to <30 mL/min/1.73 m^2^.

c eGFR < 15 mL/min/1.73 m^2^; not on dialysis therapy.

**Table 2:** Average Number of Days’ Exposure to and Cost of Study Simvastatin plus Ezetimibe per Patient, by CVD Risk and CKD Stage at Randomization

	Study Simvastatin plus Ezetimibe		Cost to Achieve LDL-C Reductions Observed in SHARP^a^
Days	Cost
By 5-year risk of CVD			
<10%	1,400 ± 18	£1,665 ± £21	£1,570 ± £20
10%-<20%	1,236 ± 17	£1,471 ± £20	£1,380 ± £19
≥20%	1,038 ± 15	£1,235 ± £18	£1,137 ± £16
By CKD stage			
3^b^	1,333 ± 18	£1,586 ± £22	£1,454 ± £20
4	1,289 ± 17	£1,534 ± £21	£1,449 ± £20
5^c^	1,064 ± 26	£1,266 ± £31	£1,182 ± £29
On dialysis	1,077 ± 18	£1,282 ± £21	£1,214 ± £20
All patients	1,200 ± 10	£1,428 ± £12	£1,319 ± £11

*Note:* Values are given as average ± standard error. Simvastatin plus ezetimibe at UK £1.19 per day (2015).

Abbreviations: CKD, chronic kidney disease; CVD, cardiovascular disease; LDL-C, low-density lipoprotein cholesterol; SHARP, Study of Heart and Renal Protection.

a Actual use of study simvastatin plus ezetimibe treatment adjusted to the use needed to achieve the LDL cholesterol level reductions observed in SHARP in the absence of other lipid-lowering treatments. For example, 57% of participants on dialysis therapy at randomization allocated to simvastatin plus ezetimibe were taking their study treatment at 2.5 years (and average study simvastatin plus ezetimibe cost for a participant on dialysis therapy at randomization was £1,282), but net use of any (including nonstudy) lipid-lowering treatment between study treatment arms at 2.5 years was 54%. Therefore, the cost of simvastatin plus ezetimibe needed to achieve the observed LDL cholesterol levels in this patient group was calculated as £1,282 × [54%/57%] = £1,214.

b 83% of participants in this category with CKD stage 3b (estimated glomerular filtration rate of 30-<45 mL/min/1.73 m^2^).

c Not on dialysis therapy.

**Table 3:** Net Cost to Avoid a Major Atherosclerotic Event with Simvastatin plus Ezetimibe in SHARP

	Major Atherosclerotic Events Avoided^a^ [A]	Simvastatin plus Ezetimibe Costs^b^ [B]	Vascular Hospital Cost Savings^b^ [C]	Net Cost per Major Atherosclerotic Event Avoided^c^ [(B-C)/(A/1,000)]
By 5-year risk of CVD				
<10%	10 (3-18)	£1,570 (£1,531-£1,609)	£78 (£19-£151)	£157,060 (£84,090-£597,940)
10%-<20%	24 (7-43)	£1,380 (£1,342-£1,417)	£141 (£34-£261)	£50,300 (£27,130-£190,060)
≥20%	58 (17-104)	£1,137 (£1,104-£1,169)	£244 (£56-£455)	£15,230 (£7,220-£64,410)
By CKD stage				
3^d^	28 (3-18)	£1,454 (£1,416-£1,492)	£113 (£28-£209)	£47,280 (£26,370-£173,760)
4	39 (11-69)	£1,449 (£1,409-£1,487)	£173 (£42-£317)	£32,020 (£17,240-£120,720)
5^e^	35 (9-67)	£1,182 (£1,125-£1,238)	£154 (£36-£302)	£29,210 (£14,170-£120,450)
On dialysis	36 (10-65)	£1,214 (£1,175-£1,253)	£193 (£44-£369)	£28,180 (£13,820-£115,380)
All patients	37 (11-65)	£1,319 (£1,298-£1,341)	£177 (£42-£326)	£30,390 (£16,050-£117,910)

*Note:* Values are given as value (95% confidence interval). Simvastatin plus ezetimibe at UK £1.19 per day (2015).

Abbreviations: CKD, chronic kidney disease; CVD, cardiovascular disease; SHARP, Study of Heart and Renal Protection.

a Per 1,000 treated for about 5 years.

b Over about 5 years per patient.

c With costs and events discounted at 3.5% per annum.

d 83% of participants in this category with CKD stage 3b (estimated glomerular filtration rate of 30-<45 mL/min/1.73 m^2^).

e Not on dialysis therapy.

**Table 4:** Projected Net Costs per QALY of 5-year LDL Lowering With High-Intensity Statin-Based Regimens Compared to no LDL Lowering in CKD

	Simvastatin plus Ezetimibe: UK £1.19/day^a^	High-Intensity^b^ Statins	
UK £0.60-£1.10/day^c^	UK £0.05-£0.10/day^c^
By 5-year risk of CVD at start of treatment			
<10%	£39,600	£30,900-£36,300	£17,300-£18,100
10%-<20%	£30,500	£24,800-£28,400	£15,800-£16,300
≥20%	£33,300	£25,300-£30,200	£12,700-£13,400
By CKD stage at start of treatment			
3^d^	£13,000	£9,100-£11,500	£3,100-£3,400
4	£22,400	£18,000-£20,800	£11,100-£11,500
5^e^	£43,300	£33,400-£39,500	£17,900-£18,800
On dialysis	£42,700	£34,000-£39,400	£20,100-£20,900

*Note:* Costs and outcomes discounted at 3.5% per annum.

Abbreviations: CKD, chronic kidney disease; CVD, cardiovascular disease; LDL, low-density lipoprotein; QALY, quality-adjusted life-year.

a Based on 20 mg simvastatin, 10 mg ezetimibe.

b Achieving ≥40% reductions in LDL cholesterol level.

c See Table S2 for high-intensity statin-based regimens in the United Kingdom in this cost range.

d 83% of participants in this category with CKD stage 3b (estimated glomerular filtration rate of 30-<45 mL/min/1.73 m^2^).

e Not on dialysis therapy.

## Supplementary Material

Supplementary Table S1 (PDF) Cox model predicting risk of a first significant vascular event.

Supplementary Table S2 (PDF) Reductions in LDL cholesterol and UK costs of different high-intensity statin-based regimens.

Supplementary Table S3 (PDF) LDL cholesterol differences at 2.5 years with use of simvastatin plus ezetimibe in SHARP.

Supplementary Table S4 (PDF) Mean hospital episodes per participant and effect of allocation to simvastatin plus ezetimibe.

Supplementary Table S5 (PDF) Number of vascular hospital episodes by case-mix group or specialty and cost per episode.

Supplementary Table S6 (PDF) Net cost per major vascular event avoided with simvastatin plus ezetimibe.

Supplementary Table S7 (PDF) Net cost to avoid a major atherosclerotic event with full adherence to simvastatin plus ezetimibe.

Supplementary Table S8 (PDF) Projected gains in QoL-adjusted survival and net cost per QALY with 5 years of simvastatin plus ezetimibe treatment.

Supplementary Figure S1 (PDF) SHARP recruitment and randomization structure.

Supplementary Figure S2 (PDF) Effect of allocation to simvastatin plus ezetimibe on all major vascular events.

Supplementary Item S1 (PDF) Supplementary methods.
