# Supplementary material for: Cost-effectiveness of Simvastatin plus Ezetimibe for Cardiovascular Prevention in CKD: Results of the Study of Heart and Renal Protection (SHARP)
Source: Am J Kidney Dis. 2016 Apr;67(4):576–84. doi: 10.1053/j.ajkd.2015.09.020 (PMC4801501; doi:10.1053/j.ajkd.2015.09.020)
Supplement: Supplementary Table S1 (PDF) — Cox model predicting risk of a first significant vascular event. [file mmc1.pdf]

Table S1: Cox proportional hazards model predicting the risk of a first significant cardiovascular event (ie, nonfatal myocardial infarction, nonfatal stroke, any revascularization procedure, or vascular death) in SHARP participants

Patient characteristic (at randomization)	Parameter category	Hazard ratio[1] (95% CI)
Gender (Reference: Female)	Male	1.4 (1.2, 1.6)
Ethnicity (Reference: White)	Asian: from China	1.3 (1.0, 1.7)
	Asian: other	0.7 (0.6, 0.9)
	Black	1.0 (0.7, 1.3)
	Other ethnicity	0.8 (0.6, 1.1)
Smoker (Reference: Never smoker)	Previous smoker	1.1 (0.9, 1.2)
	Current smoker	1.4 (1.2, 1.7)
Diabetes	Yes	1.4 (1.2, 1.7)
Systolic blood pressure, mmHg	<130	1.1 (0.9, 1.2)
(Reference: ≥130, <150)	≥150	1.3 (1.2, 1.5)
Albumin, g/dL	<3.8	1.2 (1.1, 1.4)
(Reference: ≥3.9, <4.2)	≥4.2	0.9 (0.7, 1.0)
	Missing	1.1 (0.9, 1.4)
Hemoglobin, g/dL	<11.6	1.2 (1.1, 1.4)
(Reference: ≥11.6, <13)	≥13	0.9 (0.8, 1.1)
	Missing	1.1 (0.9, 1.4)
Total cholesterol, mmol/L	<4.3	1.0 (0.9, 1.1)
(Reference: ≥4.3, <5.3)	≥5.3	1.2 (1.1, 1.4)
HDL-cholesterol, mmol/L	<0.9	1.2 (1.1, 1.3)
(Reference: : ≥0.9, <1.2)	≥1.2	1.0 (0.9, 1.1)
Urinary ACR (pre-RRT participants only), mg/g (Reference: <30)	≥30, ≤300	1.2 (1.0, 1.5)
	>300	1.4 (1.1, 1.8)
	Missing	1.2 (0.9, 1.6)
Type of renal disease (Reference: Other known / unknown cause)	Diabetic nephropathy	1.4 (1.2, 1.8)
	Cystic kidney disease	0.9 (0.7, 1.1)

**Patient characteristic (at randomization)**	**Parameter category**	**Hazard ratio[1] (95% CI)**
Age	per 10 years	1.5 (1.4, 1.6)
CKD stage at randomization (Reference: stage 3[2])	CKD stage 4	1.2 (1.1, 1.5)
	CKD stage 5	1.7 (1.4, 2.0)
	On dialysis, duration <3	2.7 (2.2, 3.5)
	On dialysis, duration >3	3.4 (2.6, 4.3)
History of vascular disease	Yes	1.9 (1.7, 2.1)

CKD, Chronic Kidney Disease;

[1]The event hazard over time was further adjusted for ezetimibe/simvastatin allocation weighted for net use of lipid lowering intervention between treatment allocation arms (a time-updated covariate in the Cox model) defined as 'net use of LDL-lowering treatment' annually during the study as the difference in the proportions of ezetimibe/simvastatin and placebo-allocated patients taking any LDL-lowering treatment (at least 80% of study ezetimibe/simvastatin or use of non-study statin). The treatment-allocation was not used in prediction of cardiovascular disease risk for the purpose of stratification into cardiovascular risk categories.

[2]83% of participants in this category with CKD stage 3b (eGFR ≥30 to <45 ml/min/1.73m^2).
