# Supplementary material for: Cost-effectiveness of Simvastatin plus Ezetimibe for Cardiovascular Prevention in CKD: Results of the Study of Heart and Renal Protection (SHARP)
Source: Am J Kidney Dis. 2016 Apr;67(4):576–84. doi: 10.1053/j.ajkd.2015.09.020 (PMC4801501; doi:10.1053/j.ajkd.2015.09.020)
Supplement: Supplementary Table S2 (PDF) — Reductions in LDL cholesterol and UK costs of different high-intensity statin-based regimens. [file mmc2.pdf]

Table S2: Reductions in LDL-cholesterol and UK costs of different high-intensity statin-based regimens

	Reduction in LDL-cholesterol (%)			
**Dose (mg/day)**	**10**	**20**	**40**	**80**
Simvastatin[1]				42%
Atorvastatin[2]		44%	48%	53%
Rosuvastatin[1]	43%	48%	53%	
Simvastatin 20mg plus ezetimibe 10mg[2]		51%		
	**Daily cost (£)[3]**			
Simvastatin				£0.07
Atorvastatin		£0.05	£0.06	£0.10
Rosuvastatin	£0.64	£0.93	£1.06	
Simvastatin 20mg plus ezetimibe 10mg		£1.19		
**LDL-cholesterol reduction potency compared to simvastatin 20mg plus ezetimibe 10mg[4]**				
Simvastatin				82%
Atorvastatin		86%	94%	104%
Rosuvastatin	84%	94%	104%	

Note: High-intensity statin as those achieving 40% or more reductions in LDL-cholesterol.

1. National Institute for Health and Care Excellence. Lipid modification: cardiovascular risk assessment and the modification of blood lipids for the primary and secondary prevention of cardiovascular disease. National Institute for Health and Care Excellence; 2014.

2. Ballantyne CM, Abate N, Yuan Z, King TR, Palmisano J. Dose-comparison study of the combination of ezetimibe and simvastatin (Vytorin) versus atorvastatin in patients with hypercholesterolemia: the Vytorin Versus Atorvastatin (VYVA) study. Am Heart J. 2005 Mar;149(3):464-73.

3. NHS Prescription Services. NHS Electronic Drug Tariff March 2015. Available from: http://www.ppa.org.uk/edt/March_2015/mindex.htm.

4. Ratio of proportional reductions in LDL-cholesterol compared to simvastatin 20mg plus ezetimibe 10mg (e.g., 0.86 for atorvastatin 20mg daily calculated as the ratio of 44% and 51%)
