# Supplementary material for: Cost-effectiveness of Simvastatin plus Ezetimibe for Cardiovascular Prevention in CKD: Results of the Study of Heart and Renal Protection (SHARP)
Source: Am J Kidney Dis. 2016 Apr;67(4):576–84. doi: 10.1053/j.ajkd.2015.09.020 (PMC4801501; doi:10.1053/j.ajkd.2015.09.020)
Supplement: Supplementary Table S3 (PDF) — LDL cholesterol differences at 2.5 years with use of simvastatin plus ezetimibe in SHARP. [file mmc3.pdf]

Table S3: LDL-cholesterol differences at 2.5 years with use of simvastatin plus ezetimibe in SHARP

	Differences between ezetimibe/simvastatin- and placebo-allocated participants		Estimated LDL-C difference (mmol/L) between ezetimibe/simvastatin- and placebo-allocated participants with full adherence to ezetimibe/simvastatin
	Net use of any LDL-C lowering treatment	LDL-C difference (mmol/L)	
**5-year risk of cardiovascular disease**			
<10%	66%	0.95	1.44
10% - 20%	61%	0.87	1.43
≥20%	58%	0.74	1.28
**CKD stage at randomization**			
3[1]	66%	0.98	1.48
4	68%	1.05	1.54
5, not on dialysis	56%	0.72	1.29
On dialysis	54%	0.60	1.11
**All patients**	**61%**	**0.85**	**1.39**

LDL-C, LDL-cholesterol; CVD, cardiovascular disease; CKD, chronic kidney disease.

[1]83% of participants in this category with CKD stage 3b (eGFR ≥30 to <45 ml/min/1.73m^2)
