# Supplementary material for: Cost-effectiveness of Simvastatin plus Ezetimibe for Cardiovascular Prevention in CKD: Results of the Study of Heart and Renal Protection (SHARP)
Source: Am J Kidney Dis. 2016 Apr;67(4):576–84. doi: 10.1053/j.ajkd.2015.09.020 (PMC4801501; doi:10.1053/j.ajkd.2015.09.020)
Supplement: Supplementary Table S4 (PDF) — Mean hospital episodes per participant and effect of allocation to simvastatin plus ezetimibe. [file mmc4.pdf]

Table S4: Mean (SD) hospital episodes per participant and effect of allocation to simvastatin plus ezetimibe in SHARP

Hospital episode category	Number of hospital episodes during SHARP		Hospital episodes per participant allocated to:		Rate ratio (95% CI)
	Ezetimibe/simvastatin n=4650	Placebo n=4620	Ezetimibe/simvastatin n=4650	Placebo n=4620	
Any atherosclerotic	873	1,029	0.19 (0.62)	0.22 (0.66)	0.84 (0.74, 0.96)
Any non-atherosclerotic vascular	953	1,070	0.20 (0.58)	0.23 (0.62)	0.89 (0.79, 0.99)
Renal	5,471	5,645	1.18 (1.95)	1.22 (1.93)	0.97 (0.90, 1.03)
Other	5,932	5,748	1.28 (1.88)	1.24 (1.77)	1.03 (0.97, 1.09)
