# Supplementary material for: Cost-effectiveness of Simvastatin plus Ezetimibe for Cardiovascular Prevention in CKD: Results of the Study of Heart and Renal Protection (SHARP)
Source: Am J Kidney Dis. 2016 Apr;67(4):576–84. doi: 10.1053/j.ajkd.2015.09.020 (PMC4801501; doi:10.1053/j.ajkd.2015.09.020)
Supplement: Supplementary Table S5 (PDF) — Number of vascular hospital episodes by case-mix group or specialty and cost per episode. [file mmc5.pdf]

Table S5: Number of vascular hospital episodes (ie, including any vascular event) in SHARP by case-mix group or specialty and cost per episode

A. Inpatient and day case admissions

HRG	Description	Number of episodes	Cost (£) per episode[1]
AA05B	Intracranial Procedures Except Trauma with Haemorrhagic Cerebrovascular Disorders - category 4 without CC	1	4,295
AA11B	Intracranial Procedures Except Trauma with Haemorrhagic Cerebrovascular Disorders - category 3 without CC	2	9,803
AA22A	Non-Transient Stroke or Cerebrovascular Accident, Nervous system infections or Encephalopathy with CC	218	2,568
AA23A	Haemorrhagic Cerebrovascular Disorders with CC	44	2,636
AA24A	Brain Tumours or Cerebral Cysts with CC	2	2,332
AA25A	Cerebral Degenerations or Miscellaneous Disorders of Nervous System with CC	4	1,727
AA26A	Muscular, Balance, Cranial or Peripheral Nerve disorders; Epilepsy; Head Injury with CC	2	1,197
AA28A	Motor Neuron Disease with CC	1	2,787
AA29A	Transient Ischaemic Attack with CC	61	909
BZ23Z	Vitreous Retinal Procedures - category 1	1	468
BZ24A	Non-Surgical Ophthalmology with length of stay 2 days or more	6	2,326
DZ09A	Pulmonary Embolus with Major CC	25	1,896
DZ09B	Pulmonary Embolus with CC	20	1,372
DZ09C	Pulmonary Embolus without CC	1	932
DZ16A	Pleural Effusion with Major CC	4	2,123
DZ17B	Respiratory Neoplasms with CC	1	1,634
DZ19A	Other Respiratory Diagnoses with Major CC	4	903
DZ21H	Chronic Obstructive Pulmonary Disease or Bronchitis without NIV without Intubation with Major CC	10	1,959

DZ23A	Bronchopneumonia with Major CC	11	2,137
DZ23B	Bronchopneumonia with CC	2	1,630
DZ24A	Inhalation Lung Injury or Foreign Body with Major CC	5	2,667
DZ26A	Pneumothorax with CC	1	1,633
DZ27D	Respiratory Failure without Intubation with Major CC	2	1,987
DZ29A	Granulomatous, Allergic Alveolitis or Autoimmune Lung Disease with CC	50	1,602
DZ29B	Granulomatous, Allergic Alveolitis or Autoimmune Lung Disease without CC	1	809
DZ51Z	Complex Tuberculosis	2	17,869
EA03Z	Pace 1 - Single chamber or Implantable Diagnostic Device	81	2,697
EA12Z	Implantation of Cardioverter - Defibrillator only	14	12,621
EA14Z	Coronary Artery Bypass Graft (First Time)	107	9,083
EA17Z	Single Cardiac Valve Procedures	63	10,851
EA19Z	Single Cardiac Valve Procedures with Percutaneous Coronary Intervention, Pacing, EP or RFA	1	12,338
EA26Z	Standard Congenital Surgery	4	4,638
EA27Z	Percutaneous Standard EP or Ablation	6	3,544
EA31Z	Percutaneous Coronary Intervention (0-2 Stents)	253	2,579
EA36A	Catheter 19 years and over	371	1,555
EA44Z	Minor Cardiac Procedures	7	2,529
EA51Z	Coronary Artery Bypass Graft with valve replacement or repair	18	11,283
EA52Z	Repair or replacement of more than one heart valve	1	12,765
EB01Z	Non interventional acquired cardiac conditions	380	721
EB02Z	Endocarditis	3	3,890
EB03H	Heart Failure or Shock with CC	9	2,309
EB03I	Heart Failure or Shock without CC	242	1,397
EB05Z	Cardiac Arrest	3	1,233
EB07H	Arrhythmia or Conduction Disorders with CC	12	1,472
EB07I	Arrhythmia or Conduction Disorders without CC	381	768

EB08H	Syncope or Collapse with CC	4	1,146
EB08I	Syncope or Collapse without CC	32	661
EB10Z	Actual or Suspected Myocardial Infarction	187	1,410
FZ02Z	Very Major Oesophageal Procedures	1	2,959
FZ05A	Major Stomach or Duodenum Procedures 2 years and over with CC	1	4,577
FZ10A	Distal colon procedures with Major CC	3	8,350
FZ10B	Distal colon procedures without Major CC	2	5,904
FZ11A	Large Intestine - Major Procedures with Major CC	12	6,764
FZ16Z	Very Major Procedures for Gastrointestinal Bleed	1	4,831
FZ17A	Abdominal Hernia Procedures 19 years and over with Major CC	1	4,531
FZ18A	Inguinal Umbilical or Femoral Hernia Repairs 19 years and over with Major CC	3	2,668
FZ18C	Inguinal Umbilical or Femoral Hernia Repairs 19 years and over without CC	1	1,506
FZ24A	Major Therapeutic Open or Endoscopic Procedures 19 years and over with Major CC	1	1,965
FZ31D	Disorders of the Oesophagus with length of stay 2 days or more with Major CC	1	2,961
FZ31E	Disorders of the Oesophagus with length of stay 2 days or more without Major CC	1	1,986
FZ36D	Intestinal Infectious Disorders with length of stay 2 days or more Major CC	3	3,597
FZ36E	Intestinal Infectious Disorders with length of stay 2 days or more without Major CC	1	1,897
FZ38D	Gastrointestinal Bleed with length of stay 2 days or more with Major CC	3	2,236
FZ38E	Gastrointestinal Bleed with length of stay 2 days or more without Major CC	1	1,398
FZ43A	Non-Malignant Stomach or Duodenum Disorders with length of stay 2 days or more with Major CC	5	2,417
FZ44A	Malignant Stomach or Duodenum Disorders with length of stay 2 days or more with Major CC	1	3,041
FZ44B	Malignant Stomach or Duodenum Disorders with length of stay 2 days or more without Major CC	1	2,371

FZ45A	Non-Malignant Large Intestinal Disorders with length of stay 2 days or more with Major CC	2	2,842
FZ45B	Non-Malignant Large Intestinal Disorders with length of stay 2 days or more without Major CC	3	1,878
FZ46A	Malignant Large Intestinal Disorders with length of stay 2 days or more with Major CC	1	3,852
FZ47A	Non-Malignant General Abdominal Disorders with length of stay 2 days or more with Major CC	9	2,863
FZ47B	Non-Malignant General Abdominal Disorders with length of stay 2 days or more without Major CC	1	1,628
FZ60Z	Diagnostic Endoscopic Procedures on the Upper GI Tract 19 years and over	1	454
FZ66A	Very Major Small Intestine Procedures 19 years and over with CC	1	7,441
GA01C	Hepatobiliary Transplant 18 years and over	1	19,620
GA10F	Open or Laparoscopic Cholecystectomy with CC	2	3,037
GC15D	Non-Malignant Liver Disorders without Major CCs	3	1,419
GC16C	Non-Malignant Pancreatic and Biliary Disorders with Major CCs	6	1,769
GC16D	Non-Malignant Pancreatic and Biliary Disorders without Major CCs	2	1,272
HA11C	Major Hip Procedures Category 2 for Trauma without CC	1	8,142
HA91Z	Hip Trauma Diagnosis without Procedure	2	2,881
HA92Z	Knee Trauma Diagnosis without Procedure	3	2,514
HA97Z	Other Trauma Diagnosis without Procedure	1	1,382
HA99Z	Other Procedures for Trauma	4	4,560
HB11A	Major Hip Procedures for non Trauma Category 2 with Major CC	1	12,106
HB11C	Major Hip Procedures for non Trauma Category 2 without CC	2	6,418
HB21A	Major Knee Procedures for non Trauma Category 2 with Major CC	1	8,764
HB21C	Major Knee Procedures for non Trauma Category 2 without CC	2	5,963

HB32A	Intermediate Foot Procedures for non -Trauma Category 2 19 years and over	5	2,538
HB34D	Minor Foot Procedures for Non -Trauma Category 2 19 years and over with CC	42	3,506
HB34E	Minor Foot Procedures for Non -Trauma Category 2 19 years and over without CC	5	1,469
HB54C	Intermediate Hand Procedures for non Trauma Category 1 without CC	3	1,498
HB99Z	Other Procedures for non Trauma	1	2,146
HC03B	Extradural Spine Intermediate 2 with CC	2	8,513
HC20C	Vertebral Column Injury without Procedure without CC	1	2,088
HC21C	Spinal Cord Injury without Procedure without CC	1	5,615
HC27B	Degenerative Spinal Conditions with CC	1	1,500
HD23A	Inflammatory Spine, Joint or Connective Tissue Disorders with Major CC	2	2,266
HD23B	Inflammatory Spine, Joint or Connective Tissue Disorders with CC	8	836
HD25A	Infections of Bones or Joints with Major CC	3	3,832
HD25B	Infections of Bones or Joints with CC	2	2,194
HR05Z	Reconstruction Procedures Category 2	2	7,171
JC03A	Major Skin Procedures category 1 with Major CC	1	6,105
JD01B	Major Skin disorders category 2 with Intermediate CC	1	1,842
JD02B	Major Skin disorders category 1 with Intermediate CC	1	1,929
JD04A	Intermediate Skin disorders category 1 with Major CC	1	2,109
JD05A	Minor Skin disorders category 2 with Major CC	1	1,368
KA03A	Parathyroid Procedures with CC	5	2,710
KA06A	Non Pituitary Neoplasia and Hypoplasia with CC	1	1,968
KA07Z	Non-Surgical Thyroid Disorders	1	1,074
KA08Z	Other Endocrine Disorders	1	1,003
KA09A	Thyroid Procedures with CC	1	3,488
KB02B	Diabetes with Hyperglycaemic Disorders 70 years and over with Intermediate CC	1	1,214

KC05A	Fluid and Electrolyte Disorders 70 years and over with Major CC	5	2,038
KC05B	Fluid and Electrolyte Disorders 70 years and over with Intermediate CC	3	1,178
KC05D	Fluid and Electrolyte Disorders 69 years and under with Major CC	6	1,836
KC05E	Fluid and Electrolyte Disorders 69 years and under with Intermediate CC	5	944
LA02A	Kidney Transplant 19 years and over from Cadaver Heart beating donor	18	19,456
LA03A	Kidney Transplant 19 years and over from Live donor	7	20,798
LA04E	Kidney or Urinary Tract Infections with length of stay 2 days or more with Intermediate CC	4	2,092
LA04F	Kidney or Urinary Tract Infections with length of stay 2 days or more without CC	2	1,518
LA07D	Acute Kidney Injury with Major CC with Interventions	1	5,111
LA07E	Acute Kidney Injury with Major CC without Interventions	4	2,234
LA07F	Acute Kidney Injury with Intermediate CC with Interventions	1	3,356
LA07G	Acute Kidney Injury with Intermediate CC without Interventions	5	1,475
LA08A	Chronic Kidney Disease with length of stay 2 days or more with Major CC	11	3,781
LA08B	Chronic Kidney Disease with length of stay 2 days or more with Intermediate CC	19	2,673
LA08C	Chronic Kidney Disease with length of stay 2 days or more without CC	8	2,827
LA09F	General Renal Disorders with length of stay 2 days or more with Intermediate CC	2	1,965
LB01A	Percutaneous Nephrostomy with CC	2	4,952
LB02A	Kidney Major Open Procedure 19 years and over with Major CC	1	8,178
LB02C	Kidney Major Open Procedure 19 years and over without CC	2	5,100

LB05C	Kidney Intermediate, Endoscopic and Percutaneous Interventions 19 years and over without CC	1	1,330
LB06D	Kidney, Urinary Tract and Prostate Neoplasms with length of stay 2 days or more with Major CC	1	3,786
LB21Z	Bladder Neck Open Procedures - Male	1	5,099
LB25B	Prostate Transurethral Resection Procedure with Intermediate CC	1	2,487
LB53Z	Scrotum, Testis or Vas Deferens Intermediate Open Procedures	1	1,875
QZ01A	Aortic or Abdominal Surgery with CC	34	8,494
QZ02A	Lower Limb Arterial Surgery with CC	65	8,088
QZ04Z	Extracranial or Upper Limb Arterial Surgery	22	4,068
QZ05A	Miscellaneous Vascular Procedures with CC	23	2,972
QZ10A	Primary Unilateral Varicose Vein Procedures with CC (includes ulceration)	7	1,256
QZ11B	Amputations without Major CC	103	9,636
QZ12Z	Foot Procedures for Diabetes or Arterial Disease, and Procedures to Amputation Stumps	14	4,035
QZ13A	Vascular Access for Renal Replacement Therapy with CC	7	1,350
QZ15B	Therapeutic Endovascular Procedures with Intermediate CC	180	3,371
QZ16B	Diagnostic Vascular Radiology and other transluminal diagnostic procedures with Intermediate CC	14	2,525
QZ16C	Diagnostic Vascular Radiology and other transluminal diagnostic procedures without CC	38	1,612
QZ17B	Non-Surgical Peripheral Vascular Disease with Intermediate CC	105	2,009
QZ20Z	Deep Vein Thrombosis	69	985
SA01D	Aplastic Anaemia with CC	1	2,194
SA13A	Single Plasma Exchange, Leucophoresis or Red Cell Exchange with length of stay 2 days or less and 19 years and over	1	527
SA31Z	Malignant Lymphoma (including Hodgkin's and non-Hodgkin's)	2	1,939
WA03V	Septicaemia with Major CC	14	2,524

WA05Q	Pyrexia of unknown origin with length of stay 5 days or more with CC	1	3,281
WA09W	Other non-viral infection with CC	6	2,070
WA11V	Poisoning, toxic, environmental and unspecified effects with Major CC	1	1,271
WA12V	Complications of Procedures with Major CC	14	3,345
WA17V	Other admissions related to neoplasms with Major CC	3	2,695
WA18V	Admission for unexplained symptoms with Major CC	2	1,440
WA21W	Other Procedures and health care problems with CC	1	918

B. Outpatient hospital episodes

NAC_HRG	Description	Number of episodes	Cost (£) per episode[2]
EA03Z	Pace 1 - Single chamber or Implantable Diagnostic Device	4	143
EA27Z	Percutaneous Standard EP or Ablation	3	247
EA36A	Catheter 19 years and over	1	206
QZ10B	Primary Unilateral Varicose Vein Procedures without CC	4	285
QZ16C	Diagnostic Vascular Radiology and other transluminal diagnostic procedures without CC	9	238

C. Further outpatient hospital episodes, by specialty

Specialty code	Hospital specialty	Number of episodes	Cost(£) per episode[3]
107	Vascular Surgery	30	133
110T	Trauma & Orthopaedics: Trauma	1	101
300	General Medicine	30	153
314	Rehabilitation	1	122
320	Cardiology	133	134
361	Nephrology	2	164
400	Neurology	3	168
Total[4]		3,904	2,667[5]

Note: Hospital episodes summarised in the table include at least one vascular event code recorded during the episode. These could be comorbidities or complications during a seemingly non-vascular HRG. Occasionally the vascular event code might not contribute directly to the mapping into HRG according the HRG grouper used.
HRG, Healthcare Resource Group; NAC_HRG, Non-admitted consultations Healthcare Resource Group; CC, Comorbidity or complication
[1]Weighted unit costs for inpatient admissions (including elective and emergency admissions and day cases) from National Schedule of Reference Costs Year: 2010-11 - NHS Trusts HRG Data
[2]National Schedule of Reference Costs Year: 2010-11 - NHS Trusts Outpatient Procedures
[3]Cost for outpatient attendance by specialty was used: National Schedule of Reference Costs Year: 2010-11 - NHS Trusts Outpatient Attendances Data.
[4]21 admitted vascular hospital episodes were not grouped into case-mix groups and their costs were imputed using mean costs for similar category of episode.
[5]Calculated as average of all vascular hospital episodes
