# Supplementary material for: Cost-effectiveness of Simvastatin plus Ezetimibe for Cardiovascular Prevention in CKD: Results of the Study of Heart and Renal Protection (SHARP)
Source: Am J Kidney Dis. 2016 Apr;67(4):576–84. doi: 10.1053/j.ajkd.2015.09.020 (PMC4801501; doi:10.1053/j.ajkd.2015.09.020)
Supplement: Supplementary Table S6 (PDF) — Net cost per major vascular event avoided with simvastatin plus ezetimibe. [file mmc6.pdf]

Table S6: Net cost per major vascular event avoided with simvastatin plus ezetimibe in SHARP

	Major vascular events avoided (per 1000 treated for about 5 years)	Net cost (£) per major vascular event avoided (95% CI)[1]
**A. Use of ezetimibe/simvastatin to achieve observed effects in SHARP**		
**5-year risk of cardiovascular disease at randomization**		
<10%	11	135,620 (74,410 to 492,880)
10% - 20%	27	44,930 (24,440 to 167,870)
≥20%	62	14,180 (6,760 to 57,110)
**CKD stage at randomization**		
3[2]	31	43,270 (24,480 to 155,990)
4	43	29,120 (15,670 to 108,940)
5, not on dialysis	39	26,010 (12,920 to 101,990)
On dialysis	38	26,350 (13,070 to 104,980)
**All patients**	**40**	**27,890 (14,770 to 106,560)**
**B. With full adherence to ezetimibe/simvastatin**		
**5-year risk of cardiovascular disease at randomization**		
<10%	16	120,800 (67,570 to 425,610)
10% - 20%	42	39,320 (22,060 to 141,750)
≥20%	102	11,140 (5230 to 44,560)
**CKD stage at randomization**		
3[2]	44	38,450 (22,380 to 134,710)
4	61	26,210 (14,450 to 95,570)
5, not on dialysis	66	21,040 (10,620 to 80,670)
On dialysis	67	20,350 (10,130 to 79,790)
**All patients**	**63**	**23,840 (12,930 to 88,960)**

CI, confidence interval; Ezetimibe/simvastatin at £1.19/day (UK, 2014); [1]with costs and events discounted at 3.5% per annum; [2]83% of participants in this category with chronic kidney disease stage 3b (eGFR ≥30 to <45 ml/min/1.73m²).
