# Supplementary material for: Cost-effectiveness of Simvastatin plus Ezetimibe for Cardiovascular Prevention in CKD: Results of the Study of Heart and Renal Protection (SHARP)
Source: Am J Kidney Dis. 2016 Apr;67(4):576–84. doi: 10.1053/j.ajkd.2015.09.020 (PMC4801501; doi:10.1053/j.ajkd.2015.09.020)
Supplement: Supplementary Table S7 (PDF) — Net cost to avoid a major atherosclerotic event with full adherence to simvastatin plus ezetimibe. [file mmc7.pdf]

Table S7: Net cost to avoid a major atherosclerotic event with full adherence to simvastatin plus ezetimibe

	Major atherosclerotic events avoided (per 1000 treated for about 5 years)	Ezetimibe/simvastatin costs (£) over about 5 years per patient	Vascular hospital cost savings (£) over about 5 years per patient	Net cost (£) per major atherosclerotic event avoided (95% CI)[1]
**5-year risk of cardiovascular disease at randomization**				
<10%	14	2,035	114	140,740 (77,320 to 519,470)
10% - 20%	38	1,906	220	44,330 (24,720 to 162,100)
≥20%	95	1,549	401	12,050 (5,650 to 50,790)
**CKD stage at randomization**				
3[2]	40	1,885	164	42,270 (24,290 to 150,060)
4	55	1,855	244	29,000 (16,140 to 105,380)
5, not on dialysis	59	1,668	262	23,800 (11,620 to 95,110)
On dialysis	63	1,732	342	21,900 (10,790 to 89,050)
**All patients**	**57**	**1,796**	**277**	**26,150 (14,180 to 98,500)**

CI, confidence interval; Ezetimibe/simvastatin at £1.19/day (UK, 2014); [1]with costs and events discounted at 3.5% per annum;

[2]83% of participants in this category with chronic kidney disease (CKD) stage 3b (eGFR ≥30 to <45 ml/min/1.73m²).
