# Supplementary material for: Cost-effectiveness of Simvastatin plus Ezetimibe for Cardiovascular Prevention in CKD: Results of the Study of Heart and Renal Protection (SHARP)
Source: Am J Kidney Dis. 2016 Apr;67(4):576–84. doi: 10.1053/j.ajkd.2015.09.020 (PMC4801501; doi:10.1053/j.ajkd.2015.09.020)
Supplement: Supplementary Table S8 (PDF) — Projected gains in QoL-adjusted survival and net cost per QALY with 5 years of simvastatin plus ezetimibe treatment. [file mmc8.pdf]

Table S8: Projected gains in quality of life-adjusted survival and net cost per quality-adjusted life year with 5 years of simvastatin plus ezetimibe treatment in SHARP

	Life years gained	Quality-adjusted life years gained	Additions cost (£) of treating end-stage renal disease	Net cost (£) per quality-adjusted life-year gained
**Simvastatin 20mg plus ezetimibe 10mg daily**				
**5-year risk of cardiovascular disease at randomization**				
<10%	0.09	0.06	1,090	39,600
10% - 20%	0.11	0.08	1,400	30,500
≥20%	0.07	0.05	810	33,300
**CKD stage at randomization**				
3[1]	0.15	0.13	440	13,000
4	0.15	0.11	1,370	22,400
5, not on dialysis	0.06	0.04	840	43,300
On dialysis	0.07	0.05	1,100	42,700

All outcomes and costs discounted at 3.5% per annum; [1]83% of participants in this category with chronic kidney disease (CKD) stage 3b (eGFR ≥30 to <45 ml/min/1.73m²)
