# Supplementary material for: Cost-effectiveness of Simvastatin plus Ezetimibe for Cardiovascular Prevention in CKD: Results of the Study of Heart and Renal Protection (SHARP)
Source: Am J Kidney Dis. 2016 Apr;67(4):576–84. doi: 10.1053/j.ajkd.2015.09.020 (PMC4801501; doi:10.1053/j.ajkd.2015.09.020)
Supplement: Supplementary Figure S1 (PDF) — SHARP recruitment and randomization structure. [file mmc9.pdf]

Figure S1: SHARP recruitment and randomization structure

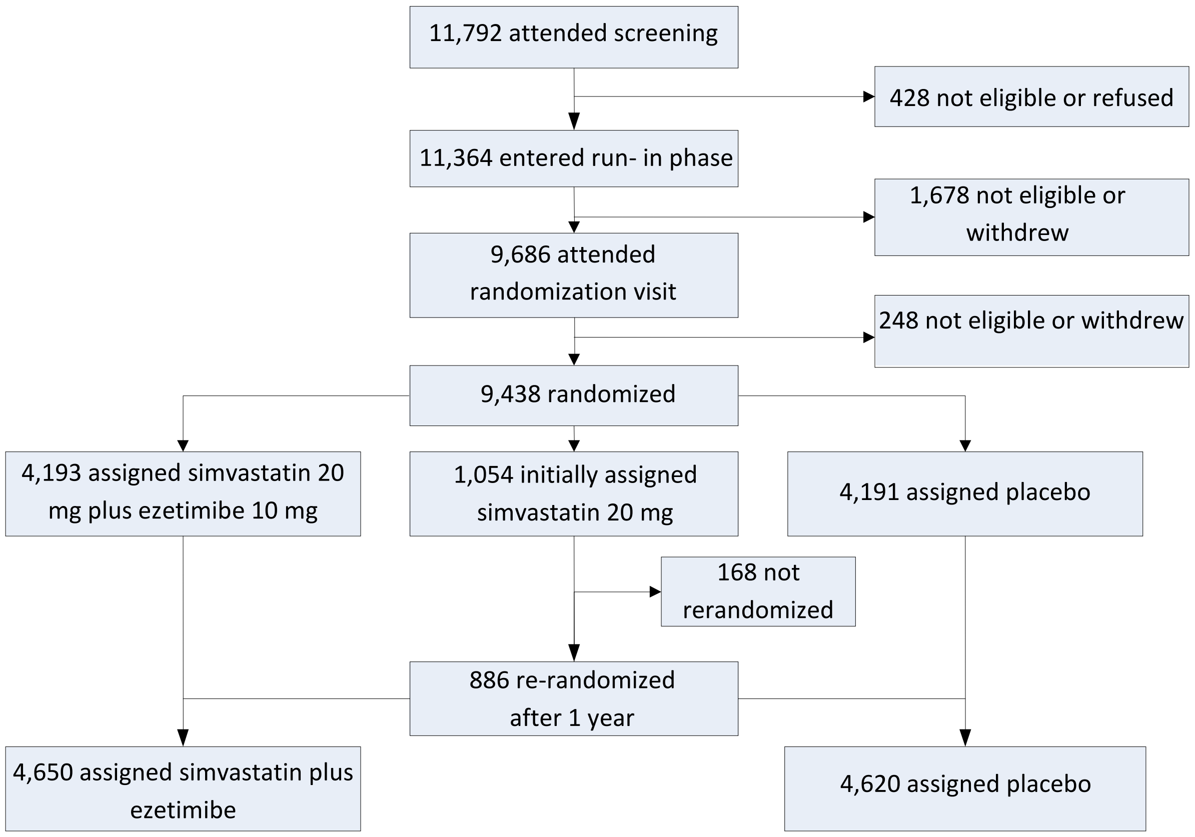

Note: At the screening visit, potentially eligible patients who agreed to participate entered a 6-week pre-randomizsation run-in period. Individuals who took at least 90% of their run-in treatment were randomizsed in a ratio of 4:4:1 between ezetimibe/simvastatin versus placebo combination versus simvastatin 20 mg daily. After 1 year, patients initially allocated simvastatin alone were re-randomizsed to ezetimibe/simvastatin versus placebo combination.
