# Supplementary material for: Cost-effectiveness of Simvastatin plus Ezetimibe for Cardiovascular Prevention in CKD: Results of the Study of Heart and Renal Protection (SHARP)
Source: Am J Kidney Dis. 2016 Apr;67(4):576–84. doi: 10.1053/j.ajkd.2015.09.020 (PMC4801501; doi:10.1053/j.ajkd.2015.09.020)
Supplement: Supplementary Figure S2 (PDF) — Effect of allocation to simvastatin plus ezetimibe on all major vascular events. [file mmc10.pdf]

Figure S2: Effect of allocation to simvastatin plus ezetimibe on all major vascular events in SHARP

	Major vascular events (first and subsequent) (annual rate per 100 participants): Simvastatin plus ezetimibe	Placebo	Rate ratio (95% CI)	Rate ratio (95% CI) per 1-mmol/L reduction in LDL-C
**By 5-year risk of CVD**				
<10%	57 (1.0)	80 (1.4)	0.70 (0.46, 1.06)	0.69 (0.44, 1.07)
10 - 20%	220 (3.3)	260 (4.0)	0.83 (0.66, 1.04)	0.81 (0.62, 1.05)
≥20%	765 (11.3)	884 (12.7)	0.89 (0.77, 1.02)	0.85 (0.70, 1.03)
**By CKD stage**				
3[1]	149 (2.9)	200 (4.1)	0.71 (0.55, 0.92)	0.71 (0.55, 0.92)
4	220 (4.0)	320 (5.5)	0.72 (0.58, 0.90)	0.73 (0.59, 0.91)
5, not on dialysis	169 (6.9)	187 (7.6)	0.90 (0.65, 1.26)	0.87 (0.55, 1.38)
**Not on dialysis**	**538 (4.1)**	**707 (5.4)**	**0.76 (0.66, 0.89)**	**0.76 (0.64, 0.89)**
**On dialysis**	**504 (8.2)**	**517 (8.6)**	**0.95 (0.79, 1.14)**	**0.92 (0.68, 1.25)**
**Overall**	**1042 (5.4)**	**1224 (6.4)**	**0.85 (0.75, 0.95) p = 0.006**	**0.82 (0.72, 0.95) p = 0.006**
Heterogeneity between CVD risk groups			$\chi^2_2 = 1.21$; $p = 0.5$	$\chi^2_2 = 0.77$; $p = 0.9$
Trend across CKD stages 3, 4, 5 and on dialysis			$\chi^2_1 = 4.85$; $p = 0.03$	$\chi^2_1 = 2.06$; $p = 0.2$
Heterogeneity between not on dialysis and on dialysis			$\chi^2_1 = 3.28$; $p = 0.07$	$\chi^2_1 = 1.29$; $p = 0.3$

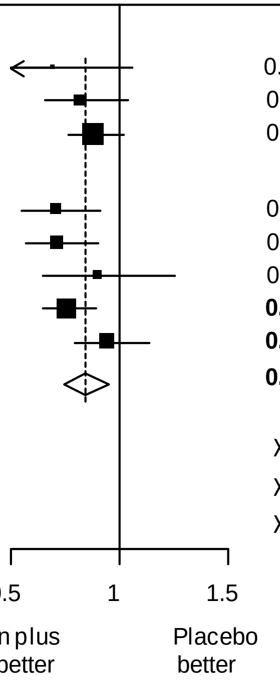

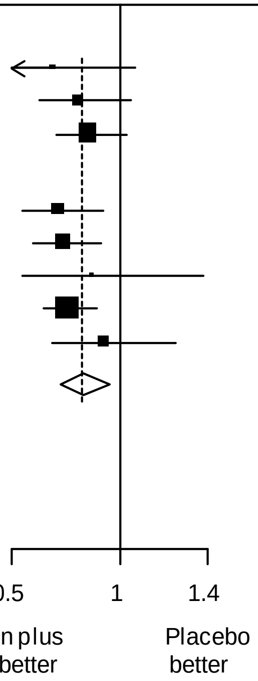

CI, confidence interval; CVD, cardiovascular disease; CKD, chronic kidney disease; LDL-C, LDL cholesterol.

[1]83% of participants in this category with chronic kidney disease (CKD) stage 3b (eGFR ≥30 to <45 ml/min/1.73m²)
