# Supplementary material for: Cost-effectiveness of Simvastatin plus Ezetimibe for Cardiovascular Prevention in CKD: Results of the Study of Heart and Renal Protection (SHARP)
Source: Am J Kidney Dis. 2016 Apr;67(4):576–84. doi: 10.1053/j.ajkd.2015.09.020 (PMC4801501; doi:10.1053/j.ajkd.2015.09.020)
Supplement: Supplementary Item S1 (PDF) — Supplementary methods. [file mmc11.pdf]

Item S1: Supplementary methods

Study design

If a participant became unwilling or unable to attend study follow-up visits, information about serious adverse events was sought from them (or their relative or carer) by telephone or from their own doctor until the scheduled end of the study. Further information was sought from hospital records and other appropriate sources about all reports of serious adverse events that might relate to study outcomes. Trained clinicians, masked to study treatment allocation, adjudicated the information in accordance with pre-specified definitions.

Assessment of health outcomes and healthcare costs

All recorded serious adverse events related to hospital care use were grouped into hospital episodes (such as hospital admissions or outpatient visits, which may have included more than one event) and, together with patient's age and disease history (including CKD stage and prior vascular disease), were mapped onto 2010-2011 UK Healthcare Resource Groups (HRGs), or onto hospital specialty for some outpatient episodes. Where no corresponding HRG or specialty could be identified (3.4% of all episodes), costs were imputed by hospital episode category and whether the episode was an admission or a day case.

Estimation of uncertainty in cost-effectiveness

A bootstrap procedure was employed to evaluate the stochastic uncertainty in cost-effectiveness results: this incorporated uncertainty in treatment effects, rates of events and hospital episodes and days on lipid lowering medication. The cost-effectiveness analysis was replicated on 5000 bootstrapped with replacement patient samples from SHARP (balanced across treatment groups) and the 95% confidence intervals for events avoided, additional costs, and cost-effectiveness were evaluated using the percentile method.

All stages of the cost-effectiveness analyses employed SHARP individual participant data, including: risk modelling and stratification into risk groups; estimation of hospital costs and ezetimibe/simvastatin costs; assessment of effects on vascular events and vascular-related hospital costs; and calculation of cost-effectiveness.

Sensitivity analyses

Sensitivity analyses assessed the cost-effectiveness of ezetimibe/simvastatin treatment under a range of assumptions about treatment costs and the level of adherence. First, we calculated results based on treatment costs ranging from the current UK ezetimibe/simvastatin price of £1.19/day to the cost of a UK generic statin regimen that produces a similar proportional reduction in LDL-cholesterol (e.g. £0.05-£0.10/day; Table S2).(1) Second, sensitivity analyses were also conducted to assess cost-effectiveness under an assumption of full adherence by calculating the subgroup-specific LDL-cholesterol difference corresponding to full adherence as the mean LDL-cholesterol reduction achieved divided by the net use of LDL-lowering treatment in the particular patient subgroup, and the cost of ezetimibe/simvastatin treatment was calculated as the follow-up time (in days) multiplied by the respective daily costs of treatment. Third, the net costs per major vascular event (MVE: major atherosclerotic event, non-coronary cardiac death [e.g. non-ischaemic heart failure, valvular heart disease and arrhythmic cardiac death] or haemorrhagic stroke) avoided were also calculated. Finally, the cost-effectiveness of ezetimibe/simvastatin among dialysis patients was also evaluated using the effects observed in SHARP within this participant subgroup (which were not independently statistically significant), to acknowledge the wider uncertainty of effects of LDL-lowering in this population.(2-4)

Long-term projections

The long-term effects of avoiding major atherosclerotic events during SHARP in categories of CKD participants were projected as follows. SHARP participants who experienced a major atherosclerotic event during the study were matched to participants not experiencing such events in a ratio 1:2 using nearest neighbour matching by their 5-year estimated cardiovascular risk.(5) Separate Gompertz proportional hazards parametric survival models were fitted to matched

participants with and without major atherosclerotic events, with cardiovascular risk, CKD stage, age at randomization, gender and region of recruitment (ie, Europe, Australia and New Zealand, China, Asia but not China and North America) as explanatory variables, and median overall survival was then calculated in each cardiovascular risk and CKD stage category. The differences between these survival times provided the projected gains in survival due to avoiding major atherosclerotic event. Annual rates of progression to end-stage renal disease (ESRD) in categories of CKD patients (among those not in ESRD at randomization) were estimated using SHARP data.(6) Quality of life in CKD stages was based on published data.(7) Costs of ESRD in the additional survival years were estimated assuming 2:1 ratio between dialysis and transplantation and respective annual costs in SHARP.(8)

All analyses were performed using R version 2.15 (www.R-project.org), SAS version 9.3 (SAS Institute Inc., Cary) or Stata 12.1 (StataCorp LP).

Work Cited

1. NHS Prescription Services. NHS Electronic Drug Tariff January 2014: Available from: http://www.ppa.org.uk/edt/January_2014/mindex.htm.
2. Palmer SC, Craig JC, Navaneethan SD, Tonelli M, Pellegrini F, Strippoli GF. Benefits and harms of statin therapy for persons with chronic kidney disease: a systematic review and meta-analysis. Ann Intern Med. 2012;157(4):263-75.
3. Upadhyay A, Earley A, Lamont JL, Haynes S, Wanner C, Balk EM. Lipid-lowering therapy in persons with chronic kidney disease: a systematic review and meta-analysis. Ann Intern Med. 2012;157(4):251-62.
4. Hou W, Lv J, Perkovic V, Yang L, Zhao N, Jardine MJ, Cass A, Zhang H, Wang H. Effect of statin therapy on cardiovascular and renal outcomes in patients with chronic kidney disease: a systematic review and meta-analysis. Eur Heart J. 2013;34(24):1807-17.
5. Becker SO, Ichino A. Estimation of average treatment effects based on propensity scores. The Stata Journal. 2002;2(4):358-77.
6. Haynes R, Lewis D, Emberson J, Reith C, Agodoa L, Cass A, Craig JC, de Zeeuw D, Feldt-Rasmussen B, Fellstrom B, Levin A, Wheeler DC, Walker R, Herrington WG, Baigent C, Landray MJ. Effects of lowering LDL cholesterol on progression of kidney disease. J Am Soc Nephrol. 2014 Aug;25(8):1825-33.
7. Erickson KF, Japa S, Owens DK, Chertow GM, Garber AM, Goldhaber-Fiebert JD. Cost-effectiveness of statins for primary cardiovascular prevention in chronic kidney disease. J Am Coll Cardiol. 2013;61(12):1250-8.
8. Kent S, Schlackow I, Lozano-Kuhne J, Reith C, Emberson J, Haynes R, Gray A, Cass A, Baigent C, Landray MJ, Herrington W, Mihaylova B. What is the impact of chronic kidney disease stage and cardiovascular disease on the annual cost of hospital care in moderate-to-severe kidney disease? BMC Nephrol. 2015;16(1):65.
